# NELARABINE-ASSOCIATED CENTRAL NERVOUS NEUROTOXICITY INDUCING GUILLAIN-BARRE-LIKE/MYELOPATHY: INPATIENT REHABILITATION AND FUNCTIONAL OUTCOMES

**DOI:** 10.2340/jrm-cc.v8.44071

**Published:** 2025-11-03

**Authors:** Rachel M. Santiago, Grace Hartl, Cono Cirone, Laura White, Erin Y. Harmon

**Affiliations:** 1 Physical Medicine and Rehabilitation, Sunnyview Rehabilitation Hospital, Schenectady, USA; 2 Neurorehabilitation Institute, Sunnyview Rehabilitation Hospital, Schenectady, USA

**Keywords:** neurological rehabilitation, neurotoxicity syndromes, rehabilitation, spinal cord diseases

## Abstract

**Objective:**

Nelarabine is an antineoplastic agent used in the treatment of acute T-cell lymphoblastic lymphoma with inadequate clinical response to prior chemotherapeutic treatments. While the most common side effects are mild to moderate haematologic events, there are known severe neurologic side effects including induced myelopathy. Due to the sparsity of cases, there is a lack of literature to guide inpatient rehabilitation programs for patients with neurological toxicity.

**Design:**

Case report.

**Patient:**

A 30-year-old woman with a history of T-cell lymphoblastic lymphoma presenting with tetraplegia after treatment with nelarabine and treated with intravenous immunoglobulin and plasma exchange therapy.

**Methods:**

After medically stabilized, she was admitted to acute inpatient rehabilitation centre with therapy program incorporating strengthening and endurance training, robot-assisted training and respiratory muscle strength training.

**Results:**

The patient demonstrated marked improvement in functional independence and manual motor testing on discharge. With ongoing participation in out-patient therapies, she achieved full independence with bed mobility and activities of daily living at a 6-month follow-up. She continues to use a wheelchair in the community, demonstrates modified independence with ambulation and is capable of climbing stairs at home.

**Conclusion:**

This case report details the functional improvements following intensive rehabilitation of a patient with severe nelarabine-associated neurotoxicity.

## LAY ABSTRACT

Nelarabine, a treatment for T-cell lymphoblastic lymphoma, can cause serious nerve damage in rare cases. This case report details the recovery of a 30-year-old woman who developed severe muscle weakness in all four limbs. Following completion of a long and intensive inpatient rehabilitation program, she regained strength and was able to return home with improved independence. Six months later, with ongoing outpatient therapies, she is still affected but has regained much of her independence in her community. Rehabilitation program details are outlined to guide rehabilitation specialists.

Nelarabine is an antineoplastic agent used for the treatment of acute T-cell lymphoblastic lymphoma with inadequate clinical response to prior chemotherapeutic treatments. Whilst the most common side effects of nelarabine are mild to moderate haematologic events, there are known severe neurologic side effects including encephalopathy and induced myelopathy (1). Encephalopathy associated with nelarabine is often reported to resolve with minor sequelae (2, 3). The current literature documenting neurological recovery of nelarabine-induced myelopathy is mixed. A study by Braish et al. demonstrated severe irreversible neurological deficits in a subset of patients, even with intravenous immunoglobulin (IVIG) and steroid treatments (4). Only a few studies have reported long-term functional outcomes, with accumulating evidence suggesting that partial or complete reversal of nelarabine-induced impairments is possible (4–8). Rehabilitation strategies are poorly documented. In this report, we discuss a unique case of nelarabine neurotoxicity causing tetraplegia. This is the first case report to our knowledge documenting short- and long-term functional outcomes after intensive multidisciplinary rehabilitation.

## CASE REPORT

A 30-year-old female with a history of T-cell lymphoblastic lymphoma, diagnosed December 2022, completed the Children’s Oncology Group AALL0434 chemotherapy regimen. This regimen consists of several therapy courses including cytarabine, daunorubicin, vincristine, pegaspargase, methotrexate and nelarabine. She underwent repeated courses of the aforementioned chemotherapeutic agents, including three cycles of nelarabine, and experienced associated acute onset of progressive encephalopathy and seizure within 24 h.

She received five sessions of IVIG and plasma exchange therapy (PLEX) for the treatment of nelarabine toxicity. She was hospitalized for 11 weeks, with hospital course notable for intubation and ventilatory support for respiratory failure secondary to neuromuscular weakness with tracheostomy placement. Magnetic resonance imaging (MRI) of the brain was unremarkable for toxic leukoencephalopathy. MRI of the cervical spine was obtained after persistent neurologic deficits were noted during acute inpatient hospitalization. Scans were revealing for intrinsic T2 cord signal abnormality involving the dorsal column with inverted V shape spanning from C1 to C2 indicative of subacute combined degeneration of the spinal cord.

She was admitted to an acute inpatient rehabilitation centre in October 2023 for approximately 9 weeks. On admission to acute rehabilitation, she presented with functional tetraplegia requiring maximum assistance for all activities of daily living (ADLs) and bed mobility. She was dependent with a Hoyer lift for transfers and was also a wheelchair dependent (Table I). Her trunk and extremities were hypotonic, and strength was significantly diminished (Table II). Sensation was absent below the C4 dermatomes, and proprioception was impaired. In addition, the patient had expressive aphasia and dysphagia requiring supplemental nutrition with a percutaneous gastrostomy tube.

**Table I:** Levels of functional independence during rehabilitation for nelarabine-associated toxicity

	Rehabilitation admission	Rehabilitation discharge	Outpatient therapy discharge
Time post-nelarabine	11 weeks	20 weeks	26 weeks
Mobility/ADL item	Level	Level	Level
Eating	NPO	Supervision/Touch	Independent
Grooming	Dependent	Supervision/Touch	Independent
Oral Hygiene	Dependent	Supervision/Touch	Independent (electric toothbrush)
Toileting	Dependent	Max Assistance	Modified Independent
Bathing	Dependent	Partial/Moderate	Modified Independent (shower seat)
Upper Extremity Dressing	Dependent	Partial/Moderate	Independent (including buttons)
Lower Extremity Dressing	Dependent	Max Assistance	Independent
Footwear	Dependent	Dependent	Modified Independent
Rolling	Max Assistance (1)	Min Assistance (1)	Independent
Supine to Sitting	Max Assistance(1)	Min Assistance (1)	Independent
Sitting to Supine	Max Assistance (1)	Min Assistance (1)	Independent
Sitting to/from Standing	Unable	Moderate Assistance (1)	Close Supervision (wheeled walker)
Transfer	Dependent (lift sling)	Min Assistance (1) SBT Moderate Assistance (1) (SPT with B AFOs)	Independent sit-sit MI SBT CS SPT with WW
Ambulation	Unable	Moderate Assistance (1) (parallel bars)	150 feet CS, MI shorter distances (WW and B AFOs)
Stairs	Unable	Unable	CG with B rails
Wheelchair mobility	Dependent	Modified Independent (ultra-lightweight chair)	Modified Independent (ultra-lightweight chair)
Car transfer	Unable	Moderate Assistance (1) SBT	Independent sit-sit, CS SPT with WW

ADL: Activities of Daily Living; WW: Wheeled Walker; SBT: Slideboard Transfer; B: Bilateral; AFO: Ankle-Foot-Orthosis; SPT: Stand Pivot Transfer; sit-sit: Sit to Sit Transfer; CS: Close Supervision; MI: Modified Independence/Independent.

(1) indicates assist of one person.

**Table II:** Manual muscle testing scores during inpatient rehabilitation

Joint/Action	Rehabilitation admission		Rehabilitation discharge	
	Left	Right	Left	Right
Shoulder flexion	1	1	4+	4+
Shoulder abduction	1	1	4+	5
Elbow flexion	0	0	5	5
Elbow extension	0	0	5	3+
Wrist flexion	0	0	4+	4+
Wrist extension	0	0	4	4
Digits	0	0	Within functional limits	Within functional limits
Hip flexion	2-	0	3-	3-
Hip extension	2-	0	2-	2-
Hip abduction	0	0	2-	2-
Hip adduction	1	0	2-	2-
Knee flexion	0	0	2-	2-
Knee extension	1	0	3	3
Ankle dorsiflexion	0	0	1	1
Ankle plantarflexion	0	0	1	1

Her barriers to rehabilitation included fluctuating hypotension and hypertension, tachycardia, visual hallucinations, anxiety and frequent suctioning for copious respiratory secretions. As she progressed, new barriers included significant neuropathic pain, musculoskeletal back pain and myoclonic tics. During acute rehabilitation, she was re-evaluated by a neurology expert for myoclonic tics with low suspicion for seizure-like activity.

Her physical therapy program included neuromuscular re-education, including neurodevelopmental positions, aquatic therapy, body-weight-supported gait training, balance training, vibratory stimulation, therapeutic activities, therapeutic strengthening exercises (passive range of motion, active-assistive range of motion and active range of motion), endurance training, functional mobility training, gait training with multiple mobility devices, power and manual wheelchair mobility training, and robotic exoskeleton gait training.

Occupational therapy focused on ADL re-education in conjunction with energy conservation and the use of compensatory techniques, pool therapy in conjunction with physical therapy, scapular mobility, upper extremity strengthening and endurance, upper extremity robot training technology and fine motor activities for the promotion of tenodesis grasps as well as anti-claw splinting.

Speech therapy utilized oral and pharyngeal exercises to target mild oral and moderate pharyngeal dysphagia present on admission. Respiratory Muscle Strength Testing (RMST) was targeted for improvement in respiratory muscle activation.

Throughout the course of rehabilitation, diet successfully progressed from inability to swallow with dependence on tube feed for nutrition to tolerating a regular consistency diet, with noted improvement on the Functional Oral Intake Scale (FOIS) from 0 to 7. The patient also experienced partial improvements in strength, sensation and proprioception. Assistance for many ADL were still required (Table I). Transfers were accomplished with the use of a slideboard and caregiver assistance. She was able to propel a lightweight wheelchair but unable to ambulate without the support of parallel bars and therapist assistance.

Upon discharge to home, she transitioned from home therapy to outpatient therapy. Her outpatient therapy program focused on vestibular balance exercise, neuromuscular exercise, dynamic gait training and stair negotiation. She progressed in her home exercise regimen to include modified rock climbing and stationary biking using modified biking shoes. At 6 months post-discharge from acute inpatient rehabilitation centre, her functional status progressed to independent with bed mobility, independent with ADL, independent with wheelchair mobility in community, modified independence with home ambulation using bilateral carbon fibre ankle-foot orthoses and wheeled walker, close supervision ambulating community distances using bilateral carbon fibre ankle-foot orthoses and wheeled walker, and contact guard assist of one person with stairs using orthotics and handrails. She returned to her previous vocation as a teacher at this time as well.

## DISCUSSION

Nelarabine-associated neurotoxicity encompasses a wide range of neurologic deficits, including post-neuropathy and myelopathy. Case reports involving severe neurologic deficits associated with nelarabine are minimal with variable outcomes. This presents challenges to the practitioner attempting to guide and council on functional prognosis. This case report documents improvement in functional outcome after participation in intensive inpatient and outpatient rehabilitation.

The patient demonstrated preferential involvement of the posterior columns within the spinal cord, which is historically associated with poor neurologic outcomes (9). On admission, she demonstrated marked functional deficits and was dependent in ADL, including grooming, oral hygiene, toileting, bathing and dressing. Our rehabilitation program focused on lower extremity strengthening, balance and proprioception training to target proprioceptive tracts within the affected dorsal-posterior columns. Additionally, robotic-assisted gait training was utilized for progressive ambulation and lower extremity strength training, which prior studies have demonstrated improvement with gait speed, balance and endurance in myelopathic cases (10). This case supports the use of robotic gait trainers for patients with myelopathy (Table I). Robot-assisted upper extremity training was also utilized with the purpose of providing a high number of repetitions to improve upper extremity range of motion, strength and cognitive awareness surrounding upper extremity movements. Robotic-assisted therapy has been shown to improve outcomes through neuroplasticity in stroke (11). Evidence for the use of upper limb robotics for spinal cord injury is less frequent but emerging (12). To our knowledge, this is the first case report demonstrating the benefit of robotic-assisted upper extremity training in a case of nelarabine neurotoxicity and myelopathy.

Barriers to our rehabilitation program included significant neuropathic pain throughout all four extremities. Pain was described as a constant discomfort associated with paresthaesia sensation exacerbated with light touch or pressure of extremity. Good response occurred with pharmacologic management with titration of gabapentin allowing continued participation and gains in therapy program. The onset of these symptoms occurred after nelarabine administration and progressed throughout the acute inpatient rehabilitation course. There is no current literature detailing neuropathic discomfort as a symptom of nelarabine toxicity nor rehabilitation response to pharmacologic management. Additional studies are needed to determine the incidence of neuropathic pain with nelarabine toxicity and the most effective strategies for pain management.

## CONCLUSION

This case study details the functional recovery trajectory and long-term outcomes of a patient with nelarabine central neurotoxicity after a specialized intensive neurologic rehabilitation program. There remains to be very few reported cases in the literature, and there is a continued need for documentation on the neurologic adverse effects and outcomes. This case serves as a guide for future therapeutic programs within the discipline of cancer and neurologic rehabilitation programs for guidance on intervention and expectations on functional and neurologic outcomes.
